# RNAseq reveals hydrophobins that are involved in the adaptation of *Aspergillus nidulans* to lignocellulose

**DOI:** 10.1186/s13068-016-0558-2

**Published:** 2016-07-19

**Authors:** Neil Andrew Brown, Laure N. A. Ries, Thaila F. Reis, Ranjith Rajendran, Renato Augusto Corrêa dos Santos, Gordon Ramage, Diego Mauricio Riaño-Pachón, Gustavo H. Goldman

**Affiliations:** Plant Biology and Crop Science, Rothamsted Research, Harpenden, Hertfordshire UK; Faculdade de Ciências Farmacêuticas de Ribeirão Preto, Universidade de São Paulo, São Paulo, Brazil; Infection and Immunity Research Group, Glasgow Dental School, School of Medicine, College of Medical, Veterinary and Life Sciences, The University of Glasgow, Glasgow, UK; Laboratório Nacional de Ciência e Tecnologia do Bioetanol (CTBE), Centro Nacional de Pesquisa em Energia e Materiais (CNPEM), Caixa Postal 6192, Campinas, SP CEP 13083-970 Brazil

**Keywords:** Fungi, Biofilm, Hydrophobin, Hydrolytic enzymes, Sugarcane bagasse

## Abstract

**Background:**

Sugarcane is one of the world’s most profitable crops. Waste steam-exploded sugarcane bagasse (SEB) is a cheap, abundant, and renewable lignocellulosic feedstock for the next-generation biofuels. In nature, fungi seldom exist as planktonic cells, similar to those found in the nutrient-rich environment created within an industrial fermenter. Instead, fungi predominantly form biofilms that allow them to thrive in hostile environments.

**Results:**

In turn, we adopted an RNA-sequencing approach to interrogate how the model fungus, *Aspergillus nidulans*, adapts to SEB, revealing the induction of carbon starvation responses and the lignocellulolytic machinery, in addition to morphological adaptations. Genetic analyses showed the importance of hydrophobins for growth on SEB. The major hydrophobin, RodA, was retained within the fungal biofilm on SEB fibres. The StuA transcription factor that regulates fungal morphology was up-regulated during growth on SEB and controlled hydrophobin gene induction. The absence of the RodA or DewC hydrophobins reduced biofilm formation. The loss of a RodA or a functional StuA reduced the retention of the hydrolytic enzymes within the vicinity of the fungus. Hence, hydrophobins promote biofilm formation on SEB, and may enhance lignocellulose utilisation via promoting a compact substrate-enzyme-fungus structure.

**Conclusion:**

This novel study highlights the importance of hydrophobins to the formation of biofilms and the efficient deconstruction of lignocellulose.

**Electronic supplementary material:**

The online version of this article (doi:10.1186/s13068-016-0558-2) contains supplementary material, which is available to authorized users.

## Background

The utilisation of sugars held within non-food, lignocellulosic, and plant biomass represents an opportunity for the development of a new generation of biofuels and green chemistries [1]. In the EU, the USA and South America wheat straw, corn stover, and sugarcane bagasse, respectively, represent abundant, renewable, and cheap lignocellulosic feedstocks applicable to green technologies. Lignocellulolytic fungi are utilised by industrial mycology for their high capacity to secrete a complex arsenal of hydrolytic enzymes that synergistically deconstruct plant cell wall polysaccharides [2]. However, efficiencies in industrial enzyme production and lignocellulose breakdown require improvement to facilitate the wide-spread implementation of such technologies [3].

In filamentous fungi, lignocellulolytic enzyme production is controlled at the transcriptional level by the competitive action of transcriptional activators and repressors [4]. In *Aspergillus nidulans*, *Trichoderma reesei*, and *Neurospora crassa*, when readily metabolizable sugars are present, such as glucose and xylose, the transcriptional repressor CreA/Cre1 blocks alternate carbon usage, including the cellulolytic and xylanolytic mechanisms and utilisation pathways [4]. Genome-wide transcriptional studies and genetic analyses have proven effective in the identification of proteins involved in the regulation of lignocelluloses deconstruction [5–8]. For example, the identification of two conserved activators of the cellulose regulon, ClrA and ClrB, in *A. nidulans* and *N. crassa* [8], and the overlapping function of the AraR and XlnR activators in the regulation of the xylanolytic pathway in *Aspergilli* [9], plus the cellulase and xylanase activators Ace2 and Xyr1 in *T. reesei* [10, 11]. Collectively, these studies have provided routes of investigation to reduce the cost of producing industrial hydrolytic enzyme cocktails required for the breakdown of plant biomass.

Filamentous fungi are not only utilised for their hydrolytic enzymes. Genomics has facilitated the discovery of metabolic enzymes and sugar transporters involved in the efficient utilisation of the breakdown products from lignocellulose deconstruction. This approach has proven particularly effective in the case of bioethanol production, where the metabolic enzymes and sugar transporters from lignocellulolytic fungi have been introduced into *Saccharomyces cerevisiae* permitting the dual uptake and fermentation of cellodextrins and xylose [12, 13]. Additional fungal proteins used in an industrial setting are hydrophobins, which self-assemble at surfaces, lowering surface water tension, adding a hydrophobic coat to the mycelia, permitting aerial growth, and promoting adhesion to surfaces, processes key to fungal sporulation, dissemination, and host infection [14, 15]. Intriguingly, hydrophobins have also been implicated at possibly playing a role in fungal deconstruction of lignocellulose. In *Aspergillus oryzae*, the hydrophobin RolA interacts with amino acid residues of the Cut1 cutinase, recruiting the enzyme to the solid–liquid interface and the surface of the polyester, polybutylene succinate-coadipate [16, 17]. In addition, an analysis of the *Aspergillus niger* transcriptome during growth in a submerged culture containing wheat straw revealed the up-regulation of hydrophobins, which was speculated to reflect their function in lignocellulolytic enzyme recruitment [5].

*Aspergillus nidulans* represents an amenable model system for the study of fungal biology and plant biomass degradation. The study of *A. nidulans* was fundamental in the formation of our current understanding of CreA-mediated carbon catabolite repression in fungi [18–20] while also representing the first filamentous fungus to demonstrate CreA nuclear-cytoplasmic shuttling in a carbon source-dependent manner [21]. In addition, the nutrient sensing pathways which regulate these downstream transcriptional mechanisms have begun to be elucidated, such as the role of central kinases, SnfA and SchA, in the regulation of CreA derepression and hydrolytic enzyme induction [21]. Recently, the extracellular mucin MsbA, which regulates mitogen-activated protein kinase signalling, was also demonstrated to influence adhesion, biofilm formation, and cellobiohydrolase A secretion [22]. The *A. nidulans* sugar uptake system is also increasingly well characterised, including the identification of high affinity hexose transporters HxtA-E, MstA, and MstC [23–25], a low affinity hexose transporter MstE [26], and the high affinity xylose transporter XtrD, which is induced in a XlnR-dependent manner and partially repressed by CreA [27]. The *A. nidulans* stress response to carbon starvation has also been well documented, permitting comparisons with the stress imposed upon the fungus during growth on lignocellulose [28]. Therefore, *A. nidulans* and the lignocellulosic feedstock, steam-exploded sugarcane bagasse (SEB) represent a highly applicable system for the study of how lignocellulolytic fungi utilise this recalcitrant plant biomass.

The present study investigated the early transcriptional response of *A. nidulans* to growth on SEB. This approach revealed how *A. nidulans* adapted to the recalcitrant, solid, carbon source, highlighting the importance of starvation responses and the induction of the xylanolytic machinery and morphological adaptations. Genetic analyses showed the importance of hydrophobins for growth on SEB. The major hydrophobin, RodA, was retained within the fungal biofilm on the lignocellulosic fibres. The StuA transcription factor that regulates fungal morphology controlled hydrophobin gene induction in response to SEB. The absence of the RodA or DewC hydrophobins reduced biofilm formation on SEB. The loss of RodA or a functional StuA facilitated the recovery of the hydrolytic enzymes from the fungal biofilm on SEB. Hence, in *A. nidulans*, hydrophobins promote biofilm formation on SEB, which may enhance lignocelluloses utilisation.

## Results

### RNA-sequencing reveals the rapid adaptation of *A. nidulans* to SEB

**Fig. 1:**
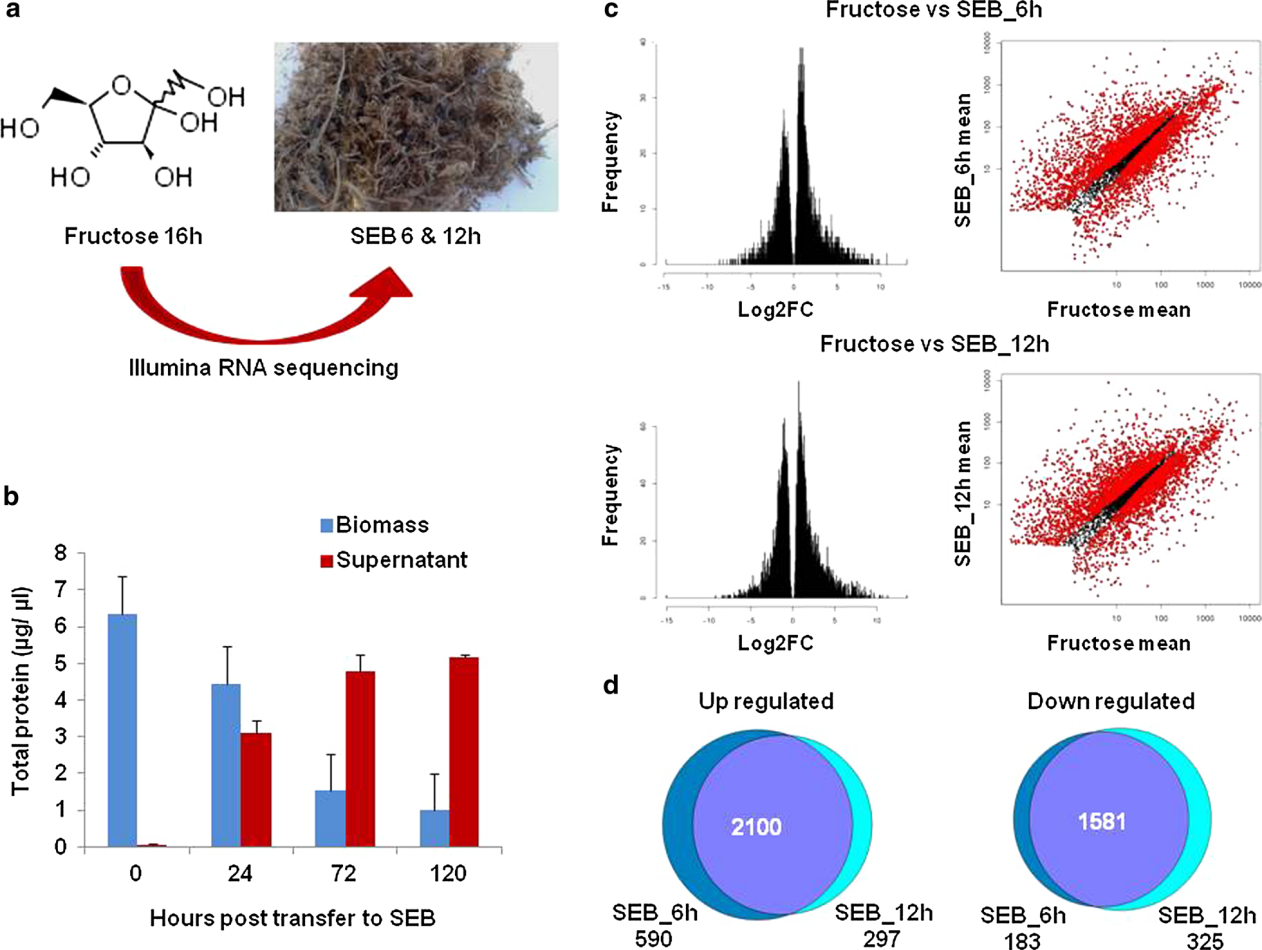
Growth of *Aspergillus nidulans* on steam-exploded sugarcane bagasse (SEB). **a** *A. nidulans* was grown in 1 % fructose liquid media for 24 h and then transferred to a semi-solid SEB media for 6–120 h at 37 °C. **b** Growth profile of *A. nidulans* grown on fructose (0 h) and post transfer to SEB for 24, 72, and 120 h shows the reduction of fungal growth and increased secretion post transfer to SEB. Presented are the mean total protein measurements of the solid and liquid fractions (plus one standard deviation) representative of fungal biomass and fungal secretion. **c** RNA-sequencing identifies the genes significantly up, or down, regulated post transfer to SEB for 6 or 12 h. **d** Venn analysis reveals a significant correlation in the modulation of transcription post 6 or 12 h growth on SEB

*Aspergillus nidulans* can grow directly on SEB as a sole carbon source in submerged culture or by solid-state fermentation (SSF). However, the growth rate is substantially slower in comparison with that on simple saccharides, such as fructose. Accordingly, when *A. nidulans* is transferred from submerged growth on fructose to SEB, the fungal biomass decreases, while the protein content in the supernatant increases (Fig. 1a, b), reflecting the significant drop in carbon availability. Subsequently, RNA-sequencing was used to interrogate how *A. nidulans* adapts to submerged growth on SEB, with the objective of identifying the early response to this recalcitrant, solid, and carbon source (Fig. 1c). The genes that were rapidly transcriptionally modulated post transfer to SEB were identified (Additional file 1: File S1), revealing 2648 and 1737 genes up- or down-regulated, respectively, after 6 h, and 2438 and 1929 genes up- or down-regulated, respectively, after 12 h. A comparison of the differentially expressed genes showed the substantial overlap in the transcriptional profiles 6 and 12 h post transfer to SEB (Fig. 1d).

Gene ontology (GO) enrichment analyses showed substantial similarities in the functional profiles of the genes up- or down-regulated post 6 or 12 h growth on SEB. Both time points demonstrated a transcriptional up-regulation of genes encoding for proteins involved in alternative carbon usage and energy production, such as autophagy, fatty acid β-oxidation, amino acid catabolism, and polysaccharide (especially xylan) catabolism (Table 1). Again, this reflected a fungal carbon starvation stress response, which promotes the utilisation of alternative intracellular carbon sources, such as lipid stores and the catabolism of cellular components via autophagy, plus the scavenging of extracellular polysaccharides. In addition, there was an up-regulation of genes encoding for proteins involved in the responses to stress, particularly DNA damage that has previously been shown to be an essential part of the *A. nidulans* carbon starvation response [28], plus the regulation of kinase activity and transcription factor activity, integral components of the signalling mechanisms to modulate such a wide-scale transcription response. Surprisingly, there was a positive regulation of genes encoding for proteins involved in asexual/sexual reproduction, sporulation and conidium formation in both time points, and the induction of cell differentiation and morphology post 12 h.

**Table 1:** Summary of the GO terms over-represented in the lists of gene up (↗) or down (↘) regulated post transfer from fructose to SEB for 6 h

GO term	Description	*p* value	Class	Reg.
Alternative carbon usage and autophagy				
GO:0019439	Aromatic compound catabolic process	0.002559	BP	↗
GO:0009083	Branched chain family amino acid catabolic process	0.000445	BP	↗
GO:0033539	Fatty acid beta-oxidation using acyl-CoA dehydrogenase	0.004303	BP	↗
GO:0045493	Xylan catabolic process	0.004047	BP	↗
GO:0044247	Cellular polysaccharide catabolic process	0.001386	BP	↗
GO:0004553	Hydrolase activity, hydrolyzing O-glycosyl compounds	0.000516	MF	↗
GO:0034727	Piecemeal microautophagy of nucleus	3.8E−05	BP	↗
GO:0000407	Pre-autophagosomal structure	0.004047	CC	↗
Sporulation				
GO:0043938	Positive regulation of sporulation	0.000876	BP	↗
GO:2000243	Positive regulation of reproductive process	0.004954	BP	↗
GO:0045597	Positive regulation of cell differentiation	0.003258	BP	↗
GO:0034305	Regulation of asexual sporulation	0.00405	BP	↗
GO:0048315	Conidium formation	0.001015	BP	↗
GO:0019953	Sexual reproduction	0.005001	BP	↗
Signal transduction and transcriptional regulation				
GO:0045859	Regulation of protein kinase activity	0.002559	BP	↗
GO:0004672	Protein kinase activity	4.65E−05	MF	↗
GO:0006355	Regulation of transcription, DNA-dependent	0.000713	BP	↗
GO:0005667	Transcription factor complex	0.005477	CC	↗
GO:0030528	Transcription regulator activity	1.47E−06	MF	↗
GO:0003700	Sequence-specific DNA binding transcription factor activity	1.39E−05	MF	↗
GO:0000077	DNA damage checkpoint	0.000601	BP	↗
GO:0042770	Signal transduction in response to DNA damage	0.000232	BP	↗
Protein translation				
GO:0000027	Ribosomal large subunit assembly	1.43E−08	BP	↘
GO:0005763	Mitochondrial small ribosomal subunit	1.35E−05	CC	↘
GO:0030687	Preribosome, large subunit precursor	4.87E−07	CC	↘
GO:0022627	Cytosolic small ribosomal subunit	2.89E−06	CC	↘
GO:0022625	Cytosolic large ribosomal subunit	4.15E−05	CC	↘
GO:0003735	Structural constituent of ribosome	5.37E−25	MF	↘
GO:0043614	Multi-eIF complex	0.004466	CC	↘
Respiration				
GO:0006096	Glycolysis	0.00425	BP	↘
GO:0015986	ATP synthesis coupled proton transport	0.00231	BP	↘
GO:0033617	Mitochondrial respiratory chain complex IV assembly	0.004466	BP	↘
GO:0042775	Mitochondrial ATP synthesis coupled electron transport	0.006022	BP	↘
GO:0033615	Mitochondrial proton-transporting ATP synthase complex assembly	0.001028	BP	↘
GO:0000275	Mitochondrial proton-transporting ATP synthase complex, catalytic core F(1)	0.004077	CC	↘
GO:0005751	Mitochondrial respiratory chain complex IV	0.0135	CC	↘
GO:0045254	Pyruvate dehydrogenase complex	0.004077	CC	↘
GO:0016491	Oxidoreductase activity	2.57E−06	MF	↘

For the full list refer to Additional file 6: File S2

*BP*,* MF*, and* CC* denote biological process, molecular function, and cellular component, respectively

Conversely, there was a down-regulation of genes encoding for proteins involved in numerous biosynthetic processes, including amino acids, coenzymes, and acetyl-CoA, accompanied by a repression of ribosome biogenesis and protein translation post 6 and 12 h growth on SEB (Table 2), which is reminiscent of a carbon starvation stress response. Accordingly, genes encoding for proteins involved in primary carbon metabolism also demonstrated a dramatic transcriptional modulation with the downregulation of glycolysis, the tricarboxylic acid cycle, the electron transport chain, oxidative phosphorylation, plus mitochondrial respiration components, such as complex IV and the ATP synthase.

**Table 2:** Summary of the GO terms over-represented in the lists of gene up (↗) or down (↘) regulated post transfer from fructose to SEB for 12 h

GO term	Description	*p*-value	Class	Reg.
Alternative carbon usage and autophagy				
GO:0034727	Piecemeal microautophagy of nucleus	0.00092	BP	↗
GO:0033539	Fatty acid beta-oxidation using acyl-CoA dehydrogenase	0.002012	BP	↗
GO:0009083	Branched chain family amino acid catabolic process	0.000123	BP	↗
GO:0019439	Aromatic compound catabolic process	0.00059	BP	↗
Sporulation				
GO:0043941	Positive regulation of sexual sporulation resulting in formation of a cellular spore	0.001234	BP	↗
GO:0075307	Positive regulation of conidium formation	0.002775	BP	↗
Signal transduction and transcriptional regulation				
GO:0031667	Response to nutrient levels	0.001154	BP	↗
GO:0005667	Transcription factor complex	0.002173	CC	↗
GO:0044212	Transcription regulatory region DNA binding	5.56E−05	MF	↗
GO:0010843	Promoter binding	5.56E−05	MF	↗
GO:0030528	Transcription regulator activity	4.54E−09	MF	↗
GO:0003700	Sequence-specific DNA binding transcription factor activity	6.17E−07	MF	↗
GO:0006468	Protein phosphorylation	0.002164	BP	↗
GO:0004674	Protein serine/threonine kinase activity	0.000797	MF	↗
GO:0042770	Signal transduction in response to DNA damage	0.00092	BP	↗
GO:0000077	DNA damage checkpoint	0.002455	BP	↗
Protein translation				
GO:0000028	Ribosomal small subunit assembly	0.002859	BP	↘
GO:0000027	Ribosomal large subunit assembly	9.06E−07	BP	↘
GO:0005763	Mitochondrial small ribosomal subunit	4.47E−05	CC	↘
GO:0022627	Cytosolic small ribosomal subunit	2.43E−06	CC	↘
GO:0022625	Cytosolic large ribosomal subunit	0.000133	CC	↘
GO:0005762	Mitochondrial large ribosomal subunit	6E−08	CC	↘
GO:0003735	Structural constituent of ribosome	9.5E−25	MF	↘
Respiration				
GO:0006007	Glucose catabolic process	0.000946	BP	↘
GO:0006099	Tricarboxylic acid cycle	0.004933	BP	↘
GO:0006119	Oxidative phosphorylation	0.000164	BP	↘
GO:0016491	Oxidoreductase activity	4.81E−05	MF	↘
GO:0045254	Pyruvate dehydrogenase complex	0.006285	CC	↘
GO:0015986	ATP synthesis coupled proton transport	0.005186	BP	↘
GO:0033617	Mitochondrial respiratory chain complex IV assembly	0.008028	BP	↘
GO:0005746	Mitochondrial respiratory chain	0.00779	CC	↘
GO:0000275	Mitochondrial proton-transporting ATP synthase complex, catalytic core F(1)	0.006285	CC	↘

For the full list refer to Additional file 7: File S3

*BP*,* MF*, and* CC* denote biological process, molecular function, and cellular component, respectively

Therefore, this analysis of the transcriptome implies that *A nidulans* down-regulates growth and protein translation while shifting metabolism to alternative carbon sources, including the use of internal, pre-existing carbon sources, and specifically inducing the synthesis of lignocellulolytic enzymes. Intriguingly, *A. nidulans* also induced processes involved in changes to hyphal morphology which are commonly associated with sporulation, such as the induction of hydrophobin encoding genes, a characteristic not observed in the submerged fungal cultures, suggesting that these processes were involved in additional functions beyond sporulation, which may be important for fungal adaptations to growth on solid lignocellulose.

### Identification of the transcription factors, CAZymes, and putative high affinity sugar transporters involved in the adaptation to SEB

**Fig. 2:**
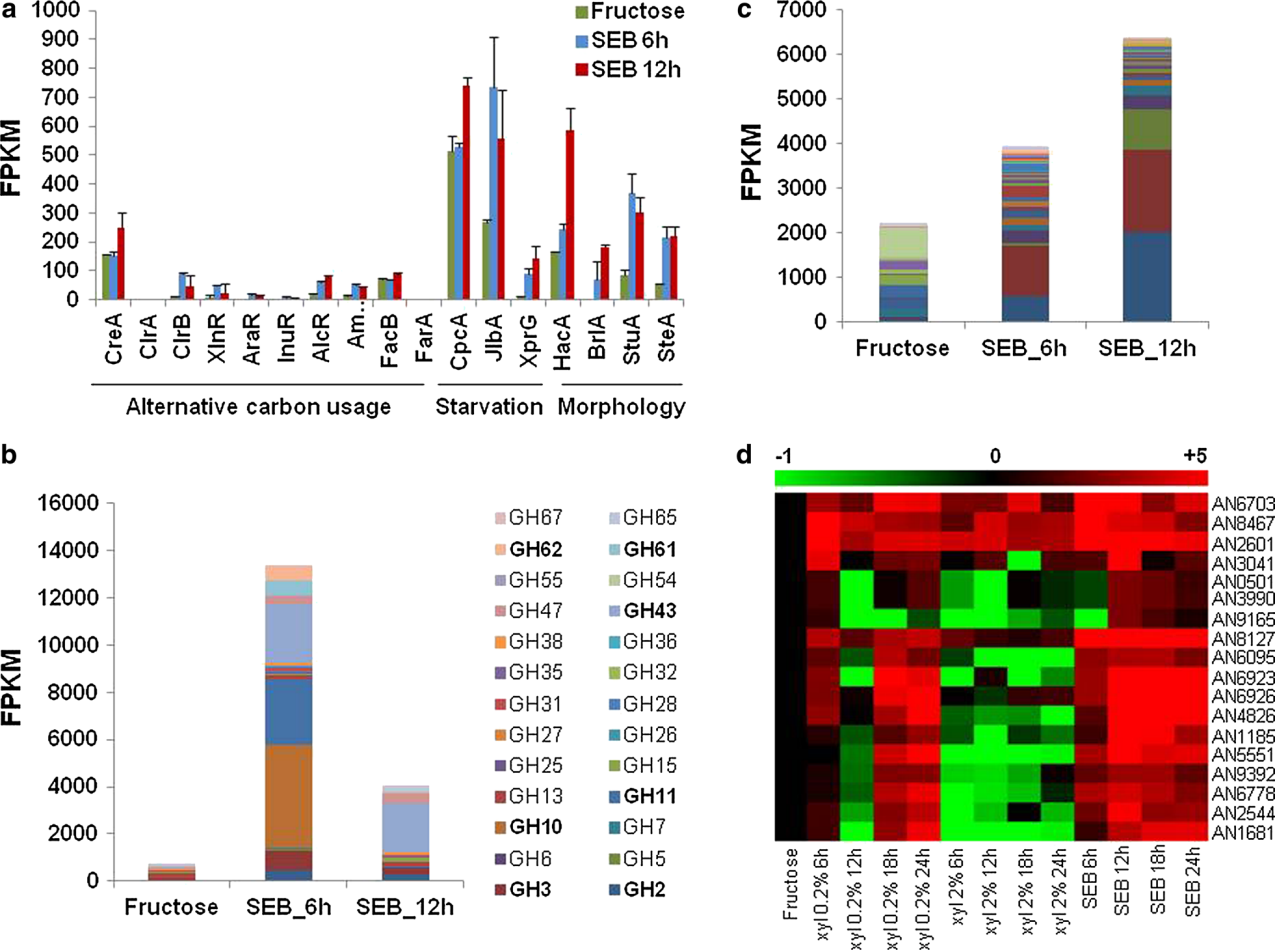
Transcriptional analyses reveal how *Aspergillus nidulans* adapts to growth on SEB. **a** Significant transcriptional modulation of genes encoding for transcription factors involved in alternative carbon usage, starvation responses, and morphological adaptations post transfer to SEB. **b** Transcriptional induction of an array of CAZymes post transfer to SEB, in particular those from the GH families which target hemicellulose GH2, GH3, GH10, GH11, GH43, GH62) or lignin (GH61 now reclassified as AA9). **c** Transcriptional induction of numerous putative and characterised sugar transporter encoding genes post transfer to SEB. **d** RT-qPCR analysis of 17 putative sugar transporter encoding genes validates RNA-seq data. A heatmap of the RT-qPCR analysis showing the expression of 17 genes during growth on 1 % fructose, 0.1 % xylose, 1 % xylose, and 0.5 % SEB. The majority of genes showed higher expression levels at low xylose concentrations implying that they encoded putative high affinity transporters

Biological processes integral to lignocellulose deconstruction and utilisation were independently assessed. Genes encoding for the transcription factors known to be involved processes highlighted in the GO analysis were identified, including the regulation of the lignocellulolytic regulon, starvation responses, alternative carbon usage, and morphology/sporulation (Fig. 2a). Transcription factors that directly regulate the lignocellulolytic regulon and alternative carbon usage or a carbon starvation response were transcriptionally induced during growth on SEB, yet despite their known importance, their absolute expression levels were low. In contrast, transcription factors involved in starvation responses and morphology/sporulation were induced to a far higher absolute level, such as morphological regulator StuA, which is required for sporulation. A broad range of hydrolytic enzymes, which predominately targeted hemicellulose (GH2, GH3, GH10, GH11, GH43, GH62) or lignin (GH61 now reclassified as AA9) were induced during growth on SEB (Fig. 2b). However, the extremely high FPKM values for two endo-xylanases (GH10: AN1818, GH11: AN3613) and an endo-arabinosidase (GH43: AN8007) implicated the specific importance of these enzymes which target the major saccharides of hemicelluloses and pectin.

Simultaneous to the enzymatic hydrolysis of SEB, *A. nidulans* is required to take up and metabolize the liberated sugars. In turn, numerous genes that encoded for putative sugar transporters were induced by transfer to SEB (Fig. 2c). The characterised high affinity hexose transporter, *hxtA* (AN6923), the low glucose sensor, *mstA* (AN8737), plus an uncharacterised putative hexose transporter (AN6804) were induced to very high levels. Characterised xylose transporters, such as *xtrA* (AN6412) and the high affinity transporter *xtrD* (AN0250), were also induced during growth on SEB. In addition, a putative transporter (AN2814) which shows homology to cellobiose transporters in *N. crassa* was induced, but at a lower absolute level. This diverse profile of putative sugar transporters reflects the heterogeneity of lignocellulose.

Subsequently, 17 putative sugar transporter encoding genes with the highest fold change in expression post transfer to SEB were selected for validation and further investigation. This independent analysis of sugar transporter gene expression confirmed the observed gene induction profile obtained via RNA-sequencing (Fig. 2d). In addition, the gene expression profiles of the putative transporter post transfer from fructose to 0.1 and 1 % xylose, implicated that the majority of the identified genes encoded putative high affinity sugar transporters. In combination with the observed induction of characterised high affinity sugar transporters, this result demonstrates how *A. nidulans* has broadly adapted the sugar uptake system to the heterogeneous, low sugar, environment encountered during growth on SEB.

Collectively, the identified, functionally characterised transcription factors and the hydrolytic arsenal induced during the early adaptation to growth on SEB are in accordance with the global GO-term functional profile of the transcriptome. These analyses highlighted the importance of alternative carbon usage in response to carbon starvation, particularly the xylanolytic regulon, in addition to the novel discovery of the potential importance of morphological adaptations, to fungal growth on solid lignocellulose.

### Fungal hydrophobins are not freely secreted and are involved in growth on SEB

The previous GO term and transcription factor analyses highlighted the participation of processes involved in morphological adaptations. In *A. nidulans*, sporulation is regulated by BrlA and StuA. Despite these transcription factors being highly induced during growth on SEB, no significant sporulation was observed in the media. Hydrophobins are small cysteine-rich, hydrophobic proteins, which can self-assemble to form monolayer at the hydrophobic:hydrophilic interface, which are involved in sporulation and are commonly found on the conidial surface [14]. In fungal pathogens, hydrophobins have also been shown to be involved in the attachment to, and penetration of, the host surface [14, 15]. In addition, hydrophobins have also been implicated as being involved in enzyme recruitment and substrate attachment in industrial mycology [16, 17].

**Fig. 3:**
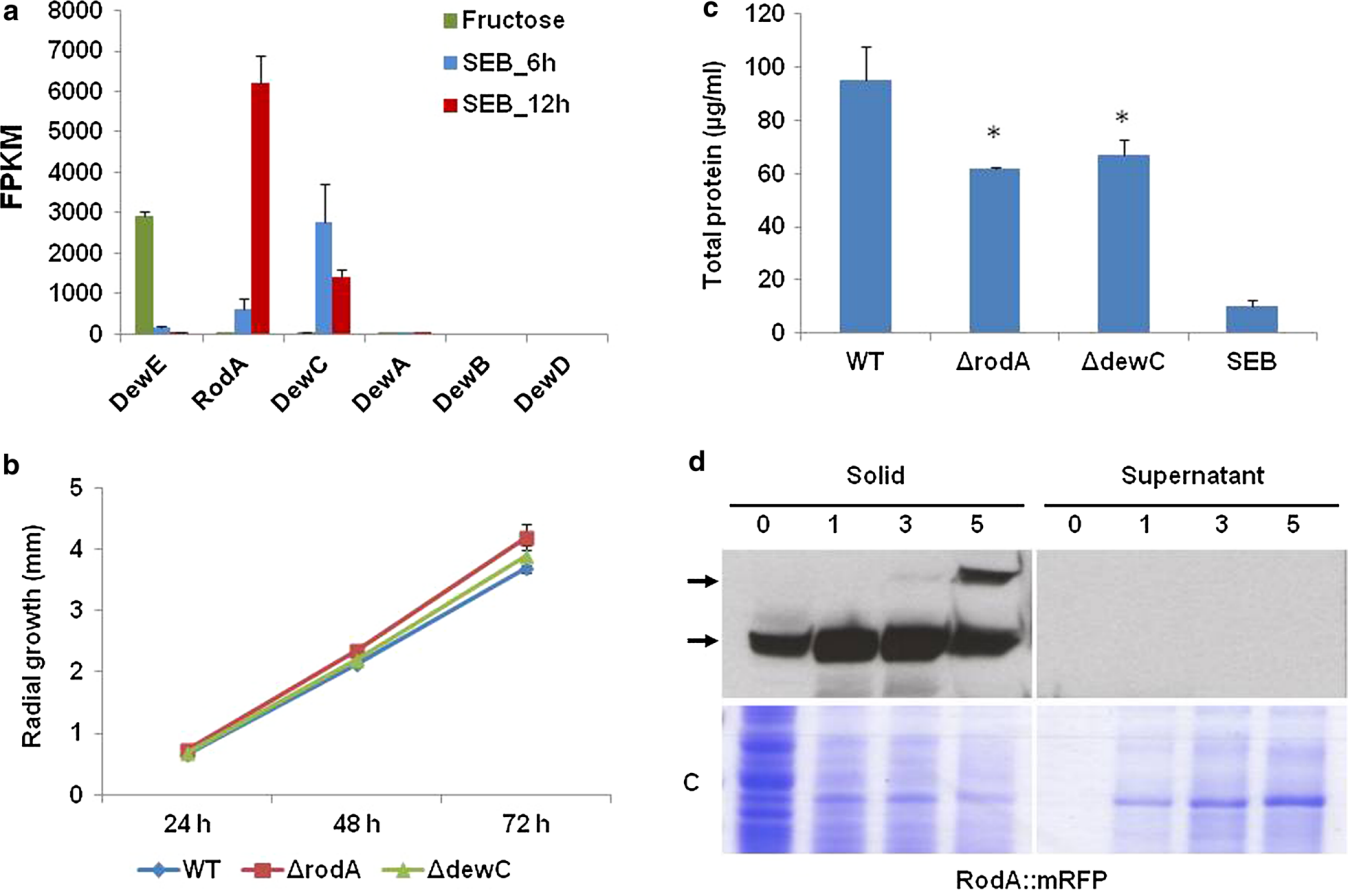
Involvement of hydrophobins in the growth of *A. nidulans* on SEB. **a** Transcriptional modulation of hydrophobin encoding genes post transfer to SEB reveals induction of *rodA* and *dewC*. **b** Radial growth on complete media is not affected by the absence of RodA or DewC. **c** Growth of *A. nidulans* on SEB is reduced in the absence of RodA or DewC. **d** Western blot showing the retention of RodA::mRFP within the solid fraction of the submerged cultivation of *A. nidulans* on SEB for 1–5 days. *Arrows* present the two RodA isoforms, potentially including a hydrophobin dimer.* C *denotes the coomassie stained 4–12 % Bis–Tris gel. Statistical significance: *p < 0.05

Inspection of all the hydrophobin encoding genes revealed the specific, high level, transcriptional induction of two hydrophobin encoding genes, *rodA* (AN8803) and *dewC* (AN6401), during growth on SEB (Fig. 3a). These two hydrophobins were also transcriptionally induced by prolonged carbon starvation [28], similar to the fungal lignocellulolytic machinery. To further investigate the importance of the identified hydrophobins for growth on SEB, *A. nidulans* strains lacking *rodA* and *dewC* were generated. The absence of RodA and DewC did not impact on radial growth on complete media (Fig. 3b). However, the hydrophobin-deficient strains demonstrated an approximate 30 % reduction (*p* < 0.05) on direct growth in submerged SEB cultures (Fig. 3c). The individual gene deletions were also complemented with the corresponding wild-type gene aiming to confirm the occurrence of possible secondary mutations during the construction of the deletion strain [28]. The corresponding re-complemented strains showed the same behaviour than the wild type with regard to their hydrophobicity and growth [28].

The hydrophobins RodA and DewC have previously been shown to localise to the surface of conidia in *A. nidulans* [29]. However, during growth on SEB, no significant conidiation was observed in the media, even after 5 or 10 days incubation. Fluorescent microscopy confirmed that RodA::mRFP localised to the conidial surface during growth in submerged glucose cultures (data not shown). However, fluorescent microscopy of RodA::mRFP during growth on SEB was impeded via the natural autofluorescence of the lignocellulose. Therefore, an alternative biochemical approach was used to evaluate the localisation of RodA::mRFP within the submerged SEB culture. The wild-type and RodA::mRFP strains were grown in glucose for 24 h and then transferred to SEB for 24, 72, and 120 h. Subsequently, the solid fraction, which contained fungal mycelia and SEB, was separated from the liquid supernatant. Total protein from the two fractions were separated via SDS Page electrophoresis and probed with the anti-mRFP antibody (Fig. 3d). No RodA::mRFP signal was detected in the solid fraction or supernatant of the wild-type strain (data not shown). The hydrophobin, RodA, was constantly detected in the solid fraction and was never detected in the supernatant of the RodA::mRFP strain, while the quantity of the RodA protein relative to the total protein content dramatically increased during growth on SEB. After 72 h growth on SEB, a second isoform of RodA appeared, potentially representing a RodA dimer. Intriguingly, trifluoroacetic acid is required to extract self-assembled hydrophobins from conidia. However, here, the extraction of RodA from mycelia was achieved with an aqueous salt solution, suggesting that during growth on SEB RodA does not form a tightly linked rod-like structure on the hyphal surface. Accordingly, the RodA monomer was predominantly detected within the solid fraction of the SEB culture.

Collectively, these results suggest that RodA is the major hydrophobin that is induced at the transcriptional and protein level during growth on SEB. The localisation of RodA in the solid fraction of the submerged SEB culture shows that RodA is retained on the mycelia or is bound to the SEB substrate. This implies that RodA either performs a role in the morphological adaptation to SEB or in enzyme retention.

### Fungal hydrophobins are required for biofilm formation on SEB

**Fig. 4:**
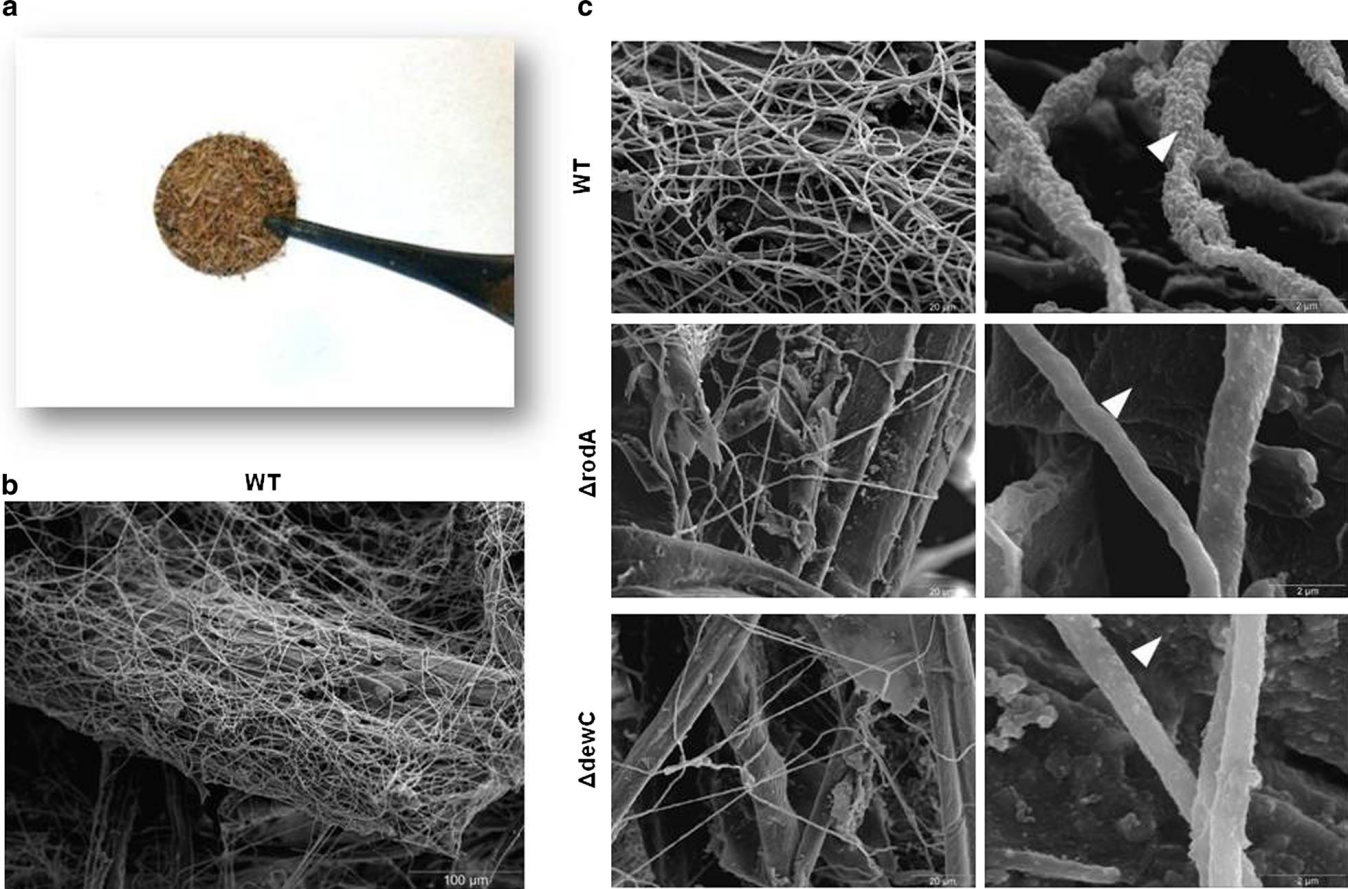
Scanning electron microscopy (SEM) of *A. nidulans* biofilms grown on SEB. **a** SEB (0.5 %) was deposited on 12 mm adhesive discs and transferred into a 24 well plate containing liquid media (without any carbon source) plus 5 × 10^5^ conidia/ml for 48 h at 37 °C. Biofilms were washed, fixed, and sputter-coated with gold prior to SEM. **b** Wild-type *A. nidulans* forms a biofilm on the SEB particles (500×). **c** Absence of RodA or DewC results in the reduction in biofilm formation on SEB (1000×) and the alteration of the appearance of the hyphal surface (12,000×)

In nature, an integral phase of fungal growth on a solid substrate is the formation of a biofilm, which is an organised heterogeneous structure that improves colony efficiencies and resistance to exogenous stresses [30]. Due to the retention of RodA to the solid fraction of the SEB culture, the importance of the two hydrophobins, RodA and DewC, to fungal biofilm formation on SEB particles within static submerged cultures was assessed via scanning electron microscopy (Fig. 4a). *Aspergillus nidulans* formed a biofilm covering the SEB particles (Fig. 4b). The formation of biofilms by the strains lacking either of the two hydrophobins was substantially reduced compared to the wild-type strain. Furthermore, a striking difference in the appearance of hyphal cell surface was found in hydrophobin mutants compared to wild-type strain (12,000× magnification), potentially representing the loss of the hydrophobin from the fungal surface (Fig. 4c). This showed that RodA and DewC were essential for growth on SEB, potentially contributing to attachment to the substrate, improving the efficiency of lignocelluloses utilisation.

### The absence of fungal hydrophobins facilitates the extraction of lignocellulolytic enzymes during the solid-state fermentation of SEB

Primarily, the absence of hydrophobins was confirmed not to have a major impact on the induction of hydrolytic enzymes during the solid-state fermentation (SSF) of SEB. This showed that the transcription of major cellulase and xylanase encoding genes, *cbhA* and *xlnA*, was not significant modulated by the absence of the hydrophobins (Additional file 2: Figure S1). However, there was a non-significant trend for the slight increased transcription of *xlnA* in the absence of DewC. Nonetheless, the hydrophobin-deficient strains did not display increased 2-deoxyglucose sensitivity, suggesting that their loss did not have a direct impact on catabolite repression and lignocellulolytic enzyme regulation.

**Fig. 5:**
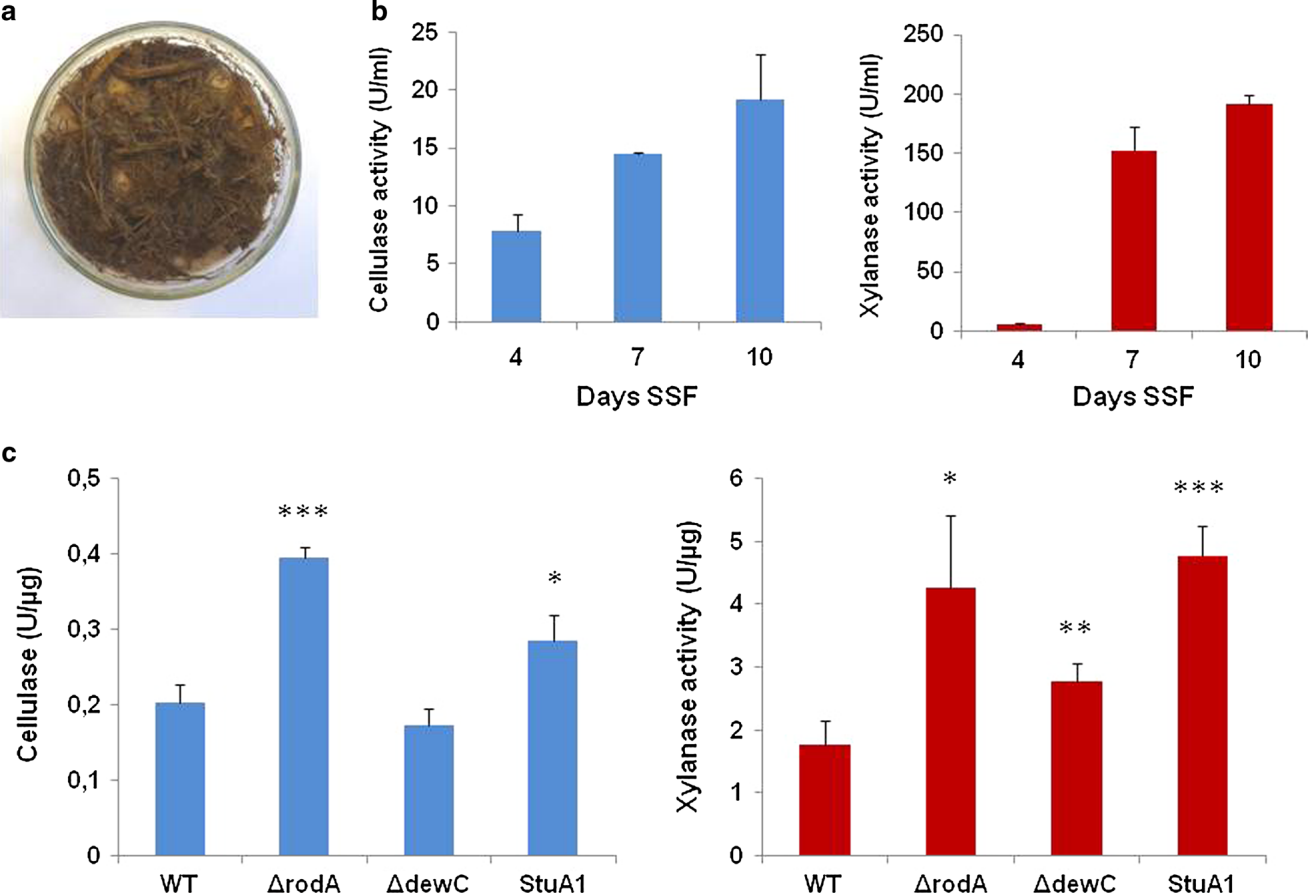
Solid-state fermentation (SSF) of *A. nidulans* on SEB. **a** Representative image of SSF. Autoclaved and dried SEB was mixed with 5 ml of liquid media without any carbon source plus 1 × 10^7^ conidia and then incubated at 37 °C for 10 days. Proteins were extracted from solid SEB cultures. The resulting supernatants were used for the respective Megazyme assays. **b** Accumulative production of cellulolytic (*blue*) and xylanolytic (*red*) enzymes by the wild-type *A. nidulans* strain throughout the 10 days SSF. **c** Recovery of lignocellulolytic enzymes (predominantly xylanases) from the *A. nidulans* biofilm was increased in the absence of RodA or DewC, or the in the presence of the non-functional StuA1 mutation, during the SSF of SEB. Enzyme activity is presented in relation to the biomass of the fungal colony. Statistical significance: *p < 0.05, **p < 0.01, ***p < 0.001

In nature, fungi form biofilms on solid substrates in a non-submerged environment, where water threatens to breakdown a compact substrate-enzyme-fungus complex. Hence, SSF can in part replicate the natural environment, where biofilms perform a role during the growth of fungi on lignocellulolytic substrata (Fig. 5a). Therefore, if fungal hydrophobins are important for the formation of a compact substrate-enzyme-fungus structure, then the extraction of hydrolytic enzymes with just water, during the SSF of SEB, should be increased by the absence of the hydrophobins. Accordingly, the proteins secreted by *A. nidulans* on the solid SEB substrate were extracted with water, representative of nature and the fungal biomass determined. This revealed the increased recovery of lignocellulolytic enzymes over time, respective to the biomass of the fungal colony, and again revealed the higher production of xylanase by the wild-type strain (Fig. 5b). Subsequently, the recovery of lignocellulolytic enzymes from the SSF culture of the different strains was assessed. Enzyme recovery from the strains lacking individual hydrophobins, especially RodA, was increased (Fig. 5c). Therefore, the absence of the hydrophobins appears increase the facility of water to extract hydrolytic enzymes from the interaction between the fungal colony and solid lignocellulose while potentially only having a minor impact on carbon catabolite repression.

### The transcription factor StuA regulates hydrophobins during semi-solid fermentation of SEB

**Fig. 6:**
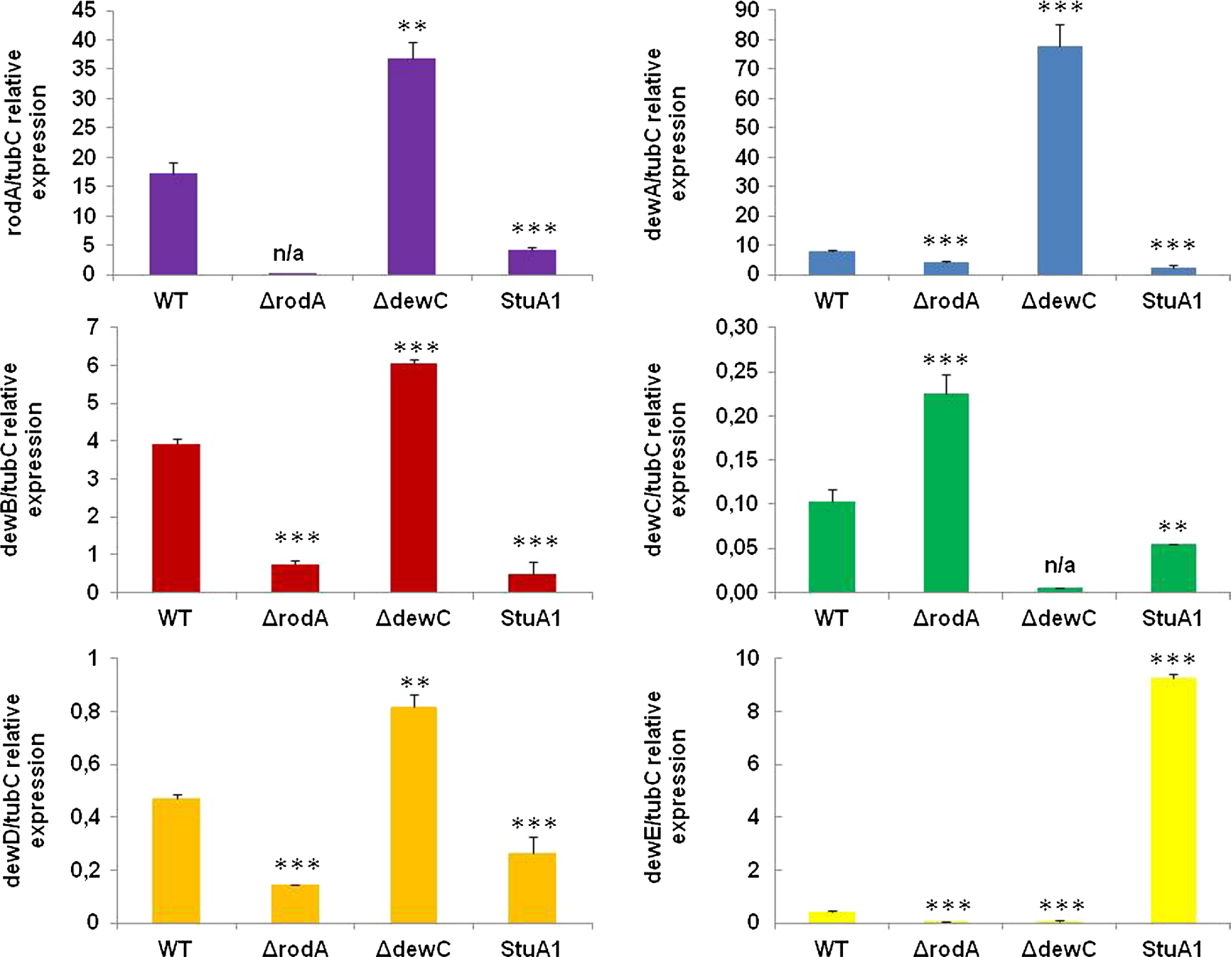
Redundancy in hydrophobin transcription during SSF of SEB. The absence of RodA, and in particular DewC, induced the transcription of alternative hydrophobin encoding genes (*rodA* and *dewA*-*E*). The regulation of multiple hydrophobin encoding genes was influenced by the presence of the non-functional StuA1 mutation. Presented is the relative expression of the hydrophobin encoding genes (cDNA hydrophobin/cDNA *tubC*) ± one standard deviation. Statistical significance: **p < 0.01, ***p < 0.001

The interplay and functional redundancy between six hydrophobins in *A. nidulans* was assessed during SSF of SEB (Fig. 6). The induction of several hydrophobins during SSF of SEB was dependent on the StuA transcription factor, which is itself induced post transfer to SEB. The absence of an individual hydrophobin resulted in the increased transcription of alternative hydrophobins. For example, the absence of RodA caused a moderate increase in the induction of *dewC* and a reduction in *dewB* and *dewD.* However, the absence of DewC resulted in a substantial increase in the induction of *rodA* and *dewA*, plus a moderate increase in the induction of *dewB* and *dewD.* Therefore, a compensatory response to the absence of DewC enables the up-regulation of alternative hydrophobins. However, compensation for the absence of RodA was insufficient, potentially contributing to the more dramatic impact of the loss of this hydrophobin on the recovery of lignocellulolytic enzymes during growth on SEB. This was also reflected in the increased recovery of lignocellulolytic enzymes from the non-functional StuA1 mutant (Fig. 5c). The expression of *cbhA* and *xlnA* was not affected in the non-functional StuA1 strain, while the expression of the hydrophobins was suggesting that the mechanism by which StuA influences lignocellulose deconstruction in *A. nidulans* was not due to alterations of carbon catabolite repression. Hence, hydrophobins promote enzyme retention and appears to be regulated by the morphological regulator StuA.

## Discussion

Sugarcane is one of the world’s most profitable crops. Waste sugarcane bagasse is a cheap, abundant, and renewable lignocellulosic feedstock for the development of the next-generation biofuels and an additional source of revenue. However, inefficiencies in the hydrolysis of SEB by microbial lignocellulolytic enzymes impede the wide-spread adoption of such green technologies. Untreated bagasse is a heterogeneous and complex solid substrate approximately consisting of 35 % cellulose, 25 % hemicellulose, and 22 % lignin [31]. Steam explosion reduces the recalcitrance of the bagasse, increasing the efficiency of enzymatic hydrolysis. Genomic comparisons of hydrolytic enzyme producers, including *Aspergillus* sp., *T. reesei*, and *N. crassa*, revealed *Aspergilli* to have a more diverse enzymatic arsenal applicable to bagasse deconstruction [32–34]. Hence, the present study documented how the model lignocellulolytic fungus *A. nidulans* rapidly adapted to growth on SEB with the objective of identifying novel processes involved in lignocellulose deconstruction.

Accordingly, the lignocellulolytic machinery of *A. nidulans* was rapidly induced post exposure to SEB, including the cellulases and xylanases, accessory proteins, such as monooxygenases, that target lignin, and a plethora of putative high affinity sugar transporters. Comparatively, the xylanase transcription and activity was far greater than the cellulases. Stress responses were induced, while protein biosynthesis was reduced. A reduction in fungal growth was accompanied by increased protein secretion. This profile reflects the decreased nutritional state of the SEB environment. The *A. niger* and *N. crassa* transcriptomes also show similarities between growth on lignocellulose and carbon starvation [5, 7]. In addition, in *A. niger*, carbon starvation induces the secretion of a subset of CAZymes, and proposed to perform a scouting function [5, 35]. In the present study, the interrogation of the early adaptations to SEB revealed that *A. nidulans* also induced morphological adaptations linked to fungal sporulation and carbon starvation, such as the two highly expressed hydrophobins, RodA, and DewC. Similarly, a previous study showed that both *rodA* and *dewC* were transcriptionally induced by prolonged carbon starvation [28]. Conversely, during asexual development in *A. nidulans*, transcripts for starvation stress responses accumulate, which is proposed to provide a protective or adaptive advantage to conidia that germinate on a substratum, where simple sugars are not freely available [36]. This includes the transcription of lignocellulolytic enzymes in the absence of their saccharide inducer molecules, such as cellobiose or xylose. Again, this shows the relationship between carbon starvation, plant biomass hydrolysis, and morphological adaptations involving hydrophobins. During growth on wheat straw, *A. niger* also induces the production of surface-interacting proteins, including the hydrophobic-binding protein, HsbA, and two hydrophobins, Hyp1 and HbD [5]. Collectively, this implies that *A. nidulans* and other fungi produce hydrophobic proteins in response to carbon starvation and growth on recalcitrant lignocellulose, which may perform roles in enzyme recruitment, substrate attachment, or altered fungal growth form.

In nature, microbes seldom exist as planktonic cells, similar to those found in the nutrient-rich liquid environment created within an experimental flask or industrial fermenter [30]. Instead, in nature, microbial biofilm communities dominate, allowing them to thrive in hostile environments [30, 37]. Organic solids function as a physical surface for microbe attachment and biofilm formation, as well as representing a source of nutrition. The adherence of a microbe to the lignocellulosic substrate is, therefore, fundamental to the establishment of a compact substrate-enzyme-microbe structure. In bacterial and fungal lignocellulolytic microbes, such as *Clostridium thermocellum*, *Streptomyces reticuli*, and *Aspergillus niger*, adhesion and biofilm formation enhances the hydrolysis of cellulose [38–40]. In *A. nidulans*, constitutive activation of the extracellular mucin receptor MsbA enhanced activation of the mitogen-activated protein kinase cascades, adhesion, biofilm formation, and cellobiohydrolase secretion during growth on cellulose, implicating the involvement of biofilm formation in cellulose deconstruction [22]. Here, the absence of the two hydrophobins RodA or DewC caused reduced biofilm formation and growth on SEB. Therefore, in *A. nidulans*, hydrophobins contribute to the formation of biofilms and fungal proliferation on lignocellulose.

The microbial biofilm creates a microenvironment which permits the establishment of hydrolysate, byproducts, and enzyme gradients. Within a biofilm, maximum fermentation rates are at the interface with the substrate, termed the reactive zone [39]. Biofilms have been shown to enhance conversion rates and the utilisation of agricultural lignocellulosic residues [38–40]. In *A. niger*, lignocellulolytic enzyme activity was higher, and fungal biomass lower, in a biofilm than in a submerged culture [40]. Energetic efficiencies favour short distances between the sites of an enzymatic reaction and the utilisation of it products. The close proximity of a lignocellulosic substrate and the secreted hydrolytic enzymes to the lignocellulolytic microbe will improve efficiencies in degradation and utilisation while also enhancing the probability that the same microbe will utilise the released saccharides. Therefore, the establishment of a compact substrate-enzyme-microbe structure, as observed in nature, is directly applicable to industrial mycology and biofuel production. Cell-associated enzymes remain in contact with the producing microbe or are retained within the extracellular matrix of a biofilm, whereas cell-free enzymes freely dissolve into the bulk environment. Therefore, microbes, such as the bacteria *C. thermocellum*, *S. reticuli*, and filamentous fungi *A. niger* and *A. nidulans*, attach to the surface of lignocellulose, permitting the formation of a biofilm and the cell-associated enzymatic hydrolysis of the lignocellulose. In fact, the disintegration of a biofilm has been associated with the release of hydrolytic enzymes into the environment [41], demonstrating the potential of a biofilm to immobilise the enzymes to the vicinity of the microbe.

SSF of lignocellulose and the production of biofilms are the most closely related experimental or industrial culturing condition to the natural fungal environment, where nutrients are more difficult to attain and more efficient hydrolytic systems are required. Here, through the utilisation of these conditions, the role of fungal hydrophobins in the deconstruction of lignocellulose was revealed. The absence of RodA or DewC in *A. nidulans* increased the recovery of hydrolytic enzymes, with just water, from the fungal biomass formed on SEB fibres, demonstrating their involvement in enzyme retention. In the Gram-negative soil dwelling bacterium *Bacillus subtilis*, the self-assembling BslA hydrophobin coats the biofilm forming a hydrophobic elastic film [42, 43]. In filamentous fungi, the production of HFBII by *T. reesei* is increased via biofilm fermentation [44], in *Pleurotus ostreatus*, the Vmh2 hydrophobin forms rodlets on the surface of the biofilm [45], and in *A. orzyae*, the RolA hydrophobin is involved in the recruitment of cutinases to the polyester, polybutylene succinate-coadipate, enhancing enzymatic targeting and improving hydrolysis [16, 17]. Therefore, the increase facility to recover lignocellulolytic enzymes from the vicinity of *A. nidulans* during SSF of SEB caused by the absence of RodA or DewC may reflect the breakdown of the integrity of the fungal biofilm and deficiencies in the ability to repel water [29], which in nature may promote a close substrate-enzyme-microbe structure, enhancing efficiencies in lignocellulose utilisation.

The study of *A. niger* grown on wheat straw proposed a model for the XlnR-independent regulation of accessory proteins, such as hydrophobin, via a mechanism beyond the lignocellulolytic regulon [5]. In *Penicillium decumbens*, BrlA associates with the expression of cellulases [46]. In *A. nidulans*, the transcription factors, BrlA and StuA, regulate fungal morphology and sporulation [47, 48]. The formation of the hydrophobic rodlet layer on the conidium of *A. nidulans* and the transcription of RodA is regulated by BrlA [47, 48]. Here, both BrlA and StuA were induced post transfer of *A. nidulans* to SEB. The induction of RodA during SSF of SEB was impeded via the introduction of a non-functional StuA1 mutation, demonstrating the potential role of the BrlA-StuA pathway in the morphological adaptation of *A. nidulans* to SEB.

## Conclusion

Collectively, this study revealed the importance of morphological adaptations by the fungus to growth on lignocellulose. The production of fungal hydrophobins was pivotal to the formation of a fungal biofilm on SEB fibres, which may promote a close substrate-enzyme-microbe structure and repel water, enhancing efficiencies in secreted enzyme retention and lignocelluloses utilisation. This novel study highlights the importance of hydrophobin during the deconstruction of lignocellulose, potentially providing new avenues to improve industrial efficiencies, applicable to numerous green technologies. The inclusion of hydrophobins within hydrolytic cocktails or the construction of hydrophobin-enzyme fusion proteins may, therefore, improve enzyme targeting to the interface with the solid lignocellulosic substrate, enhancing enzymatic activity and the release of fermentable sugars for industrial fermentation.

## Methods

### Strains and culture conditions

The *A. nidulans* wild-type strain, R21, was used as reference in all experiments. The genotypes of all the other genetically modified strains utilised in this investigation are listed in Additional file 3: Table S1. All strains were propagated at 37 °C in minimal media (1 % w/v glucose, nitrate salts, trace elements, pH 6.5) or complete media (YG: 2 % w/v glucose, 0.5 % w/v yeast extracts, trace elements) plus or minus agar (2 % w/v), in addition to the respective supplements depending on the strains auxotrophy.

### Media shift experiments and enzyme activity assays

For media shift experiments, 1 × 10^7^ conidia were inoculated into minimal media plus 1 % fructose (50 ml) and incubated on a rotary shaker (180 rpm) set at 37 °C for 24 h. Transferred mycelia was washed with sterile water, resuspended in liquid minimal media plus 0.5 % SEB, and incubated under the same conditions for 6, 12, 24, 72, and 120 h. Cultures were filtered and the mycelia frozen in liquid nitrogen prior to being freeze dried for RNA and/or protein extraction. Supernatants from the 120 h cultures were collected for endo- cellulase or xylanase activity assays (Megazyme). The fungal biomass within the SEB cultures cannot be measured directly, due to the presence of plant biomass. Therefore, total protein content was used as a relative measurement.

### RNA extraction

Fungal biomass was harvested at the presented intervals and immediately frozen in liquid nitrogen. Total RNA was extracted using TRIzol (Invitrogen), treated with DNAse (Promega) and purified using RNeasy Plant Mini Kit (Qiagen). RNA integrity was confirmed using the Bioanalyser Nano kit (Agilent technologies) and the Agilent Bioanalyser 2100.

### Protein extraction

The solid fraction of the culture containing mycelia and SEB was ground in liquid nitrogen and added immediately to the protein extraction buffer [Tris base pH 7.5 25 mM, EGTA pH 7.5 15 mM, MnCl_2_ 15 mM, plus a protease inhibitor cocktail (Roche)], vortexed for 5 min prior to centrifugation for 15 min at 14,000*g*. In addition, to obtain an insight into the protein content of the culture supernatant, the liquid fraction (40 ml) was freeze dried and resuspended in protein extraction buffer. Protein content of the solid and liquid fractions was subsequently measured using the Bio-Rad protein assay.

### Library preparation and RNA-sequencing

RNA-sequencing libraries were prepared using the Illumina Truseq v2 kit with polyA-based mRNA enrichment. Each condition was evaluated with three biological replicates. Sequencing was carried out in a HiSeq 2000 using unstranded, paired-end (2 × 50 bp) chemistry. Quality analyses were performed using FastQC (http://www.bioinformatics.babraham.ac.uk/projects/fastqc/). Short reads were cleaned using Trimmomatic [49], removing low-quality sequences and TruSeq adaptors. High quality, short reads were mapped to the *A. nidulans* genome downloaded from *Aspergillus* Genome Database (http://www.aspdg.org) [50] using Bowtie2 [51] and Tophat2 [52]. Sequencing depth was evaluated with RSeQC [53]. The rRNAs were identified with ITSx [54] and extracted prior to mapping. The RNA-seq data were submitted as raw fastq files to the NCBI Short Read Archive (SRA), accession number SRP063062 associated with BioProject PRJNA294437.

*Aspergillus nidulans* transcripts were assembled using Cufflinks [55], which also normalised transcript expression to fragments per kilobase of transcript per million mapped fragments (FPKM). Tabulated FPKM values for transcripts in each sample were obtained using Cuffcompare. A Perl script was used to filter out transcripts that were not expressed (two replicates with zero FPKM). Differentially expressed genes were identified with NOIseqBIO, which implements a non-parametric empirical Bayes approach to call differentially expressed genes while controlling for high false discovery rates, using a probability threshold of 0.95 [56, 57]. Gene ontology (GO) enrichment analyses of the significantly modulated gene sets were performed using FetGOat [58] with a critical FDR *p* value of 0.05, a minimal annotation group size of 2, and the Benjamini and Hochberg correction.

### RT-qPCRs validation of RNA-seq

All putative sugar transporter encoding genes, according to the bioinformatic prediction of the Aspergillus genome database, were identified within the RNA-seq data set. The 17 putative sugar transporter encoding genes with the highest fold change in expression, post transfer to SEB, were selected for validation via RT-qPCR. Media shift experiments were performed as described. Minimal media was inoculated with 1 × 10^7^ conidia for 24 h. Subsequently, the mycelia was washed and transferred to minimal media plus 0.5 % SEB or 0.2 and 2 % xylose. Total RNA was extracted and purified as previously described. cDNA was synthesised from 5 µg of RNA using SuperScript III (Invitrogen). Quantitative PCR (qPCR) analyses were performed according to Semighini et al. [59]. The abundance of the respective mRNAs was normalised for fungal biomass using *tubC.* The primers for the investigated genes are listed in Additional file 4: Table S2.

### Fungal transformations

The gene replacement cassette for *dewC* (AN6401) was obtained from the Fungal Genetic Stock Centre (http://www.FGSC.net). Briefly, the 5′ and 3′ gene-specific flanks were amplified from *A. nidulans* genomic DNA. Primers, 5′ reverse and 3′ forward, had 5′ extensions complementary to the *pyrG*^*Af*^ cassette, whereas 5′ forward and 3′ reverse had 5′ extensions complementary to the vector. Co-transformation of *S. cerevisiae* with the 5′ and 3′ flanks together with the *pyrG*^*Af*^ cassette and a *S. cerevisiae* vector generated a plasmid containing the gene-specific deletion construct [60]. The final linear deletion construct was PCR-amplified from the *S. cerevisiae* DNA with primers external primers. A total of 20 µg from purified cassette was used for *A. nidulans* transformations (TN02A3) according to Osmani et al. [61]. Transformants were scored for their ability to grow on minimal medium without uridine and uracil, and were then checked by PCR and Southern blot analysis [62] (Additional file 5: Fig. S2). The pre-existing Δ*rodA* and the generated Δ*dewC* strains with opposing auxotrophies were sexually crossed. The progeny of a sexual cross was screened for the absence of the hydrophobin encoding gene and the presence of the deletion cassette via PCR. The primers used are listed in Additional file 4: Table S2.

### Evaluation of fungal growth and biofilm formation

Radial growth on solid, complete media agar plates was assessed after 4 days growth at 37 °C. To evaluate growth rate on SEB under submerged conditions, 1 × 10^7^ conidia were inoculated directly into 0.5 % SEB 50 ml minimal media and incubated on a rotary shaker (180 rpm) set at 37 °C for 10 days. The mycelia and solid SEB were filtered from the cultures, frozen in liquid nitrogen and freeze dried prior to the determination of total protein content via the Bio-Rad protein assay.

For scanning electron microscopy (SEM), representative biofilms were grown on SEB discs as previously described [63]. Briefly, SEB (0.5 %) was deposited on 12 mm adhesive discs (Agar scientific, UK) and transferred into a 24 well plate for biofilm formation. SEB discs were incubated in minimal media (without any carbon source), with 5 × 10^5^ conidia/ml, for 48 h at 37 °C. Biofilms were washed with PBS before fixation in 2 % paraformaldehyde, 2 % glutaraldehyde and 0.15 % (w/v) alcian blue in 0.15 M sodium cacodylate (pH 7.4). The samples were sputter-coated with gold and viewed under a JEOL JSM-6400 scanning electron microscope (JEOL (UK) Ltd., UK) in high vacuum mode at 10 kV.

### Western blots

The wild-type (R21) and *rodA(p)::mRFP::rodA* strains (1 × 10^7^ conidia) were grown 50 ml liquid minimal media plus 1 % fructose at 37 °C in a rotatory shaker (180 rpm) for 16 h. After, the mycelia were transferred to SEB media for 24, 72, and 120 h. The collected mycelia and SEB were immediately frozen and the proteins extracted as previously described. Culture supernatants (40 ml) were freeze dried and resuspended in extraction buffer. Protein content was measured using the Bio-Rad protein assay. Total proteins (20 µg) were separated on a Bolt^®^ 4–12 % Bis–Tris Plus Gel and transferred onto a nitrocellulose membrane using the iBlot^®^2 dry blotting system (Life Technologies). Coomassie and ponceau stained gels revealed protein loading and correct transfer [62]. The primary anti-RFP (ABcam: AB65856) was used at a 1:1000 dilution in TBS-T (25 mM Tris, 0.15 M NaCl, 0.05 % Tween-20, pH 7.5) overnight at 4 °C. Primary antibodies were detected using a horseradish peroxidase (HRP)-conjugated second antibody (Kirkegaard and Perry Laboratories) at a 1:2000 dilution in TBS-T plus 5 % skimmed milk powder for 1 h, at room temperature. Chemiluminescence was detected using Super signal West Pico chemiluminescent substrate (Pierce).

### Solid fermentation experiments

Five ml of minimal medium without any carbon source was inoculated with 1 × 10^7^ conidia of the respective fungal strain and added to 0.5 g SEB (autoclaved and dried) in a small plate, mixed thoroughly, and incubated at 37 °C for 10 days. To extract the enzymes, 10 ml of ice cold ddH_2_O was mixed with the solid SEB culture and incubated on ice for 4 h with vortexing every 30 min. The supernatant was separated from the solid fraction by filtering through miracloth. Samples were centrifuged for 2 min to remove debris. The resulting supernatants were used to perform the respective enzymatic assays (Megazymes).

### Statistics

Three biological replicates were performed for all experiments and the statistical tests for significance determined via a one-tailed *t* test unless stated otherwise.

## Additional files

10.1186/s13068-016-0558-2 The *A. nidulans* genes transcriptionally modulated post transfer from fructose to SEB.
10.1186/s13068-016-0558-2 The absence of hydrophobins has minor influence on hydrolytic enzyme transcription. The transcription of* cbhA* and* xlnA* during SSF of SEB was moderately increased in the individual absence of RodA or DewC.
10.1186/s13068-016-0558-2 A list of* A.nidulans* strains used in this investigation.
10.1186/s13068-016-0558-2 A list of the primers used in this investigation.
10.1186/s13068-016-0558-2 Southern blot confirmation of Δ*dewC*. Genomic DNA from* A. nidulans* wild type, Δ*dewC* were isolated and cleaved with the enzyme* Hind*III; a 1-kb DNA fragment from the 5′-noncoding region was used as a hybridization probe. This fragment recognizes a single DNA band (about 5.3-kb) in the wild type strain and a single DNA band (about 2.5-kb) in the Δ*dewC*.
10.1186/s13068-016-0558-2 The GO terms over-represented in the lists of gene up or down regulated post transfer from fructose to SEB for 6 h.
10.1186/s13068-016-0558-2 The GO terms over-represented in the lists of gene up or down regulated post transfer from fructose to SEB for 12 h.

## Abbreviations

CAZyme: carbohydrate-active enzyme
FPKM: fragments per kilobase of transcript per million mapped reads
GH: glycoside hydrolase
GO: gene ontology
SEB: steam-exploded sugarcane bagasse
SSF: solid state fermentation
