# Supplementary figures and images for: RNAseq reveals hydrophobins that are involved in the adaptation of Aspergillus nidulans to lignocellulose
Source: Biotechnol Biofuels. 2016 Jul 19;9:145. doi: 10.1186/s13068-016-0558-2 (PMC4950808; doi:10.1186/s13068-016-0558-2)

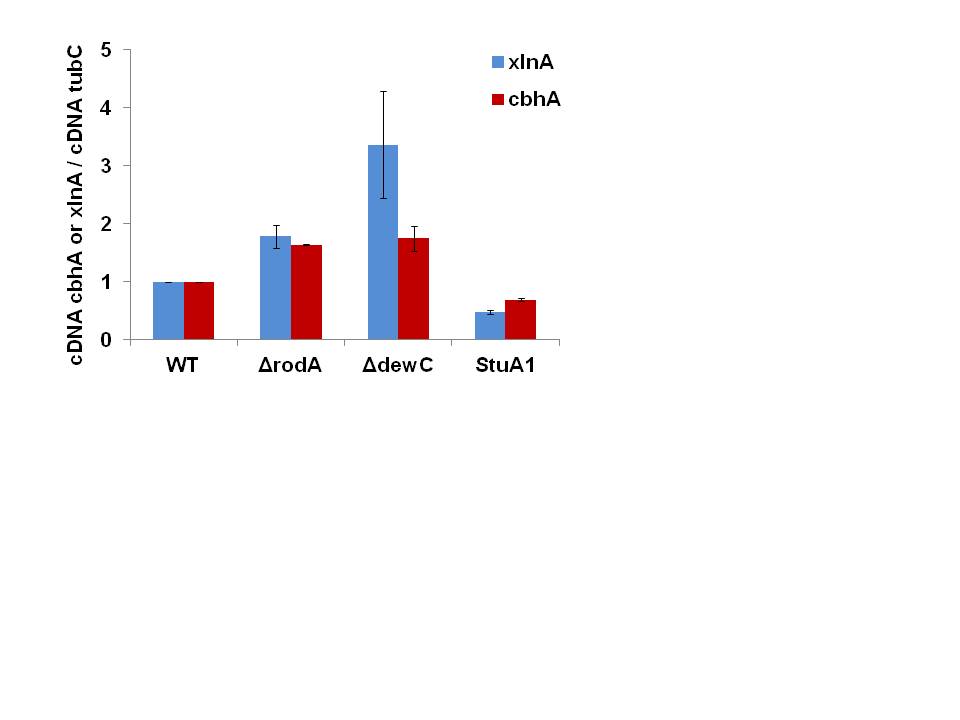

Supplement: Supplementary file 2 — 10.1186/s13068-016-0558-2 The absence of hydrophobins has minor influence on hydrolytic enzyme transcription. The transcription of cbhA and xlnA during SSF of SEB was moderately increased in the individual absence of RodA or DewC. [file 13068_2016_558_MOESM2_ESM.jpg]

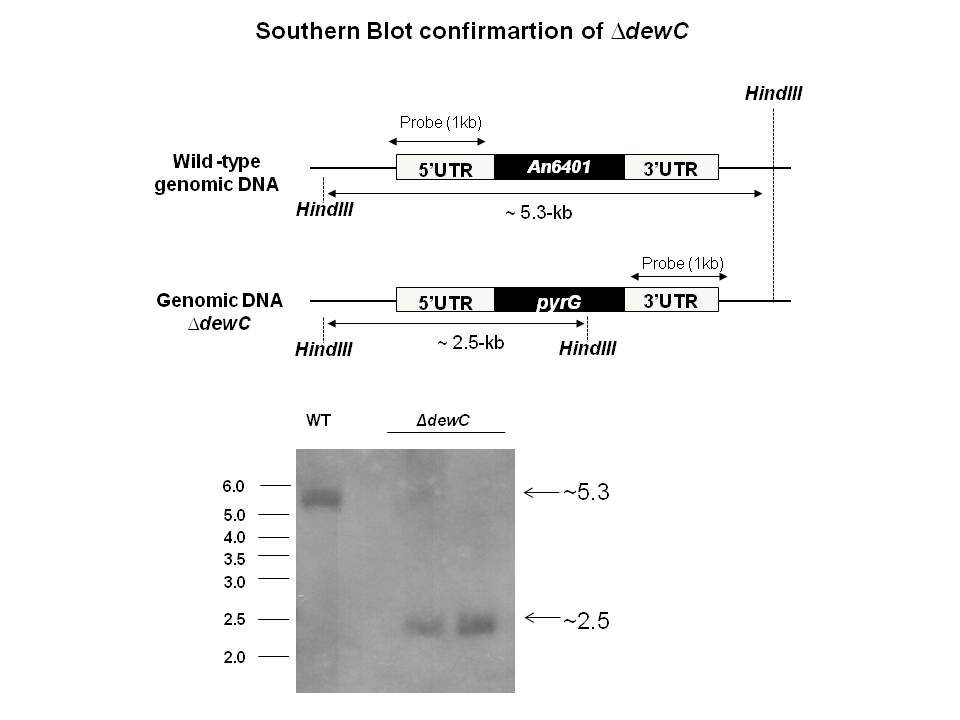

Supplement: Supplementary file 5 — 10.1186/s13068-016-0558-2 Southern blot confirmation of ΔdewC. Genomic DNA from A. nidulans wild type, ΔdewC were isolated and cleaved with the enzyme HindIII; a 1-kb DNA fragment from the 5′-noncoding region was used as a hybridization probe. This fragment recognizes a single DNA band (about 5.3-kb) in the wild type strain and a single DNA band (about 2.5-kb) in the ΔdewC. [file 13068_2016_558_MOESM5_ESM.jpg]
