# Supplementary material for: RNAseq reveals hydrophobins that are involved in the adaptation of Aspergillus nidulans to lignocellulose
Source: Biotechnol Biofuels. 2016 Jul 19;9:145. doi: 10.1186/s13068-016-0558-2 (PMC4950808; doi:10.1186/s13068-016-0558-2)
Supplement: Supplementary file 3 — 10.1186/s13068-016-0558-2 A list of A.nidulans strains used in this investigation. [file 13068_2016_558_MOESM3_ESM.docx]

**Supplementary Table S1** A list of *A.nidulans* strains used in this investigation.

| **Strain** | **Genotype** | **Origin** |
| --- | --- | --- |
| **TN02A3** | *pyrG89; pyroA4; nkuA::argB* | FGSC |
| **R21** | *pabaA4; yA1* | FGSC |
| **Δ*rodA*** | *pabaA1 yA2; ΔargB::trpC; ΔrodA::argB; veA1 trpC801* | FGSC A849^a^ |
| **RodA::mRFP** | *pyrG89; pyroA4; nkuA::argB; rodA(p)::mRFP::rodA; pyrG^Af^* | R. Fischer ^b^ |
| **Δ*dewC*** | *pyrG89; pyroA4; nkuA::argB, ΔdewC:: pyrG^Af^* | This study |
| **StuA1** | *biA1;stuA1* | FGSC A584 ^c^ |

^a^ Stringer, M.A., Dean, R.A., Sewall, T.C. & Timberlake, W.E. Rodletless, a new Aspergillus developmental mutant induced by directed gene inactivation. *Genes Dev.* **5(7)**, 1161-71 (1991).

^b^ Grünbacher, A., Throm, T., Seidel, C., Gutt, B., Röhrig, J., Strunk, T., Vincze, P., Walheim, S., Schimmel, T., Wenzel, W. & Fischer, R. Six hydrophobins are involved in hydrophobin rodlet formation in *Aspergillus nidulans* and contribute to hydrophobicity of the spore surface*. PLoS One*. 9(4), e94546 (2014).

^c^ Miller, K.Y., Toennis, T.M., Adams, T.H. & Miller, B.L. Isolation and transcriptional characterization of a morphological modifier: the *Aspergillus nidulans* stunted (stuA) gene. *Mol Gen Genet.* **227(2)**, 285-92 (1991).
