# Supplementary material for: RNAseq reveals hydrophobins that are involved in the adaptation of Aspergillus nidulans to lignocellulose
Source: Biotechnol Biofuels. 2016 Jul 19;9:145. doi: 10.1186/s13068-016-0558-2 (PMC4950808; doi:10.1186/s13068-016-0558-2)
Supplement: Supplementary file 4 — 10.1186/s13068-016-0558-2 A list of the primers used in this investigation. [file 13068_2016_558_MOESM4_ESM.docx]

**Supplementary Table S2** A list of the primers used in this investigation.

| **Primer** | **Sequence (5’- 3’)** |
| --- | --- |
| **5’UTR dewC Fw** | GTAACGCCAGGGTTTTCCCAGTCACGACGAGGCACTGAAGAGAGGATAC |
| **3’ UTR dewC Rv** | CTATCCTCCTCACATCGTTCGCTGTTTCCTGTGTGAAATTGTTATCCGC |
| **5’UTR dewC insert check Fw** | GCTGAGGCACTGAAGAGAGG |
| **pyrG Rv** | GAATTCGCCTCAAACAATGCTCTTCACC |
| **rodA 5UTR Fw** | CTCGAAGCTCTGTTCCTTGC |
| **rodA 3UTR Rv** | TTCATAGGGGCATTCTGAGG |
| **argB Rv** | TCGTTTGCTGAAGATCATGG |
| **rodA Fw** | AGCAACGTCAAGTTCCCTGT |
| **rodA Rv** | TGGAGCACTGATCGAAGAGA |
| **dewA Fw** | ATGCGCTTCATCGTCTCTCT |
| **dewA Rv** | AGCAGACCGCTCAACAGACT |
| **dewB Fw** | GCCGAAGGAAGCTCTAAGTG |
| **dewB Rv** | AGCAACCGCTGAAAGCAC |
| **dewC Fw** | CACAATCGCTTCCCTTATCG |
| **dewC Rv** | AGACCCTCCTCTTCCTCCTG |
| **dewD Fw** | CTTTCCACCTCCGCTGCT |
| **dewD Rv** | TGACTCCCTCAAACGAGACC |
| **dewE Fw** | CCACTGCCCTCTCTGTTCTC |
| **dewE Rv** | ATTGCAGCTGCGTCGATAC |
| **xlnA Fw** | ATCAACTACGGCGGAAG |
| **xlnA Rv** | CAGTAATAGAAGCCGACCC |
| **cbhA Fw** | CCCCAACTCTGAGTCCA |
| **cbhA Rv** | TCAGACTCAACAACATCAGG |
| **AN2544 Fw** | ACGCAGTTTGCCTACATTGC |
| **AN2544 Rv** | GCACAATAAGCACAAGATAG |
| **AN3041 Fw** | CCATGATATAGAGTTCTTCG |
| **AN3041 Rv** | GCTTGATGTGGACGGCCAGT |
| **AN6923 Fw** | CGCTCTTTGTTCCGCCACTT |
| **AN6923 Rv** | CGGGAGCTGATCTTCCTTTT |
| **AN4826 Fw** | GAACAGTCCCGTTGAAGACG |
| **AN4826 Rv** | CATCATAAAGCCGGCCATAT |
| **AN1185 Fw** | TTGTCGTGGTTCTCCTCTGC |
| **AN1185 Rv** | ACCCTATGAAAAACATGGCG |
| **AN8760 Fw** | CGTCTCATCCTTCGTTGCGT |
| **AN8760 Rv** | TCTAGGTTGGAGAGGGAGGC |
| **AN1681 Fw** | GGAGGAGAAAAAGAAGGCAA |
| **AN1681 Rv** | CGTAAGGGGGCACGGTCATG |
| **AN8127 Fw** | CGAACCTACACGCATCCCTG |
| **AN8127 Rv** | GTCCTACGACGAATAGAGCG |
| **AN9165 Fw** | GCATGGCGTCGAGGAGAAGA |
| **AN9165 Rv** | CCTTGAGGACAAACAGGAAT |
| **AN6804 Fw** | CCTCAACCTGATAGCCTGGG |
| **AN6804 Rv** | CCTGATAAAGTACGGCAGCC |
| **AN6095 Fw** | GGCTACCTCTCCCAGTTTGT |
| **AN6095 Rv** | GTGACCATGATGCCCGTTGC |
| **AN9392 Fw** | GCATTCTGGTCTGGAACTCG |
| **AN9392 Rv** | CATACTTGGAGTACTTGCCG |
| **AN6703 Fw** | GCTCAAGAGGCATTGGTTGC |
| **AN6703 Rv** | ACCTGGGTCACTGTTACGGC |
| **AN8467 Fw** | GCTGGTAATGAACATCGCCG |
| **AN8467 Rv** | TACTGCTTCTTTTTCACGGC |
| **AN6926 Fw** | GGGTCGATCTCCGTCGTGAT |
| **AN6926 Rv** | CGTTCCATCCCCTTTCTCCT |
| **AN5551 Fw** | CGGAGATTACCTGCCAACCA |
| **AN5551 Rv** | GCTGTTGATGCCCAGGGTCT |
| **AN0501 Fw** | ATCACAGTAGCCAAGTTTGG |
| **AN0501 Rv** | GCGTTTTAGCTCGGAGTTGC |
| **AN2601 Fw** | GCTACCGCACATTGATTGTG |
| **AN2601 Rv** | GGGCAGAAGTGATGAGACCG |
| **AN6778 Fw** | TACCAGGCAAACAACATCCG |
| **AN6778 Rv** | CAGAAGTAAAGACTCAAAGC |
| **AN3990 Fw** | GGAATCTTGCGGGTCTATAT |
| **AN3990 Rv** | GCATTAACATCCAACCCCAT |
| **tubC Fw** | AGCTGGCGGTAACAAATACG |
| **tubC Rv** | ACCTGATCCACCAATTCTGC |
